# Oral Submucous Fibrosis: A Review of the Current Concepts in Management

**DOI:** 10.7759/cureus.47259

**Published:** 2023-10-18

**Authors:** Avneet K Chhabra, Ravikant Sune, Amit Reche

**Affiliations:** 1 Dentistry, Sharad Pawar Dental College and Hospital, Datta Meghe Institute of Higher Education and Research, Wardha, IND; 2 Oral Medicine and Radiology, Sharad Pawar Dental College and Hospital, Datta Meghe Institute of Higher Education and Research, Wardha, IND; 3 Public Health Dentistry, Sharad Pawar Dental College and Hospital, Datta Meghe Institute of Higher Education and Research, Wardha, IND

**Keywords:** pentoxifyllin, aloe vera, management, precancerous disorder, oral submucous fibrosis

## Abstract

Oral submucous fibrosis (OSMF) is a precancerous disorder of the submucosa that causes inflammation and progressive fibrosis, leading to pronounced stiffness and trismus. Chewing betel nuts is a significant risk factor for OSMF in India. Arecoline from betel nuts and copper, which causes fibroblast dysfunction and the development of fibrotic bands, are the main components of betel quid. OSMF is distinguished by fibrosis in the submucosal region, which affects the majority of the oral cavity and results in advanced lockjaw due to rigidity in the lips, pharynx, cheeks, and upper third of the oesophageal canal, which progresses to dysphagia. The prevalence of OSMF is rising, particularly among younger generations, as more commercially available areca nut products like gutka (chewing tobacco) and others are being introduced. The severity of OSMF develops as the practice continues and is permanent. It also persists even after chewing has been stopped. The hallmark of oral submucous fibrosis (OSF) is abnormal collagen deposition. It is a precancerous condition and progresses to malignant tumours. Symptoms include ulcers, xerostomia, submucous fibrosis, burning sensation, and a reduction in mouth opening. Each of these drastically reduces the patient's quality of life. In the past, many treatment modalities have been tried but none of them has resulted in a cure for the disease. The primary focus of the treatment is to reduce the signs and symptoms so that the patient can have a better quality of life. Along with principles, conservative, medical, and surgical management issues have also been covered.

## Introduction and background

**Figure 1:**
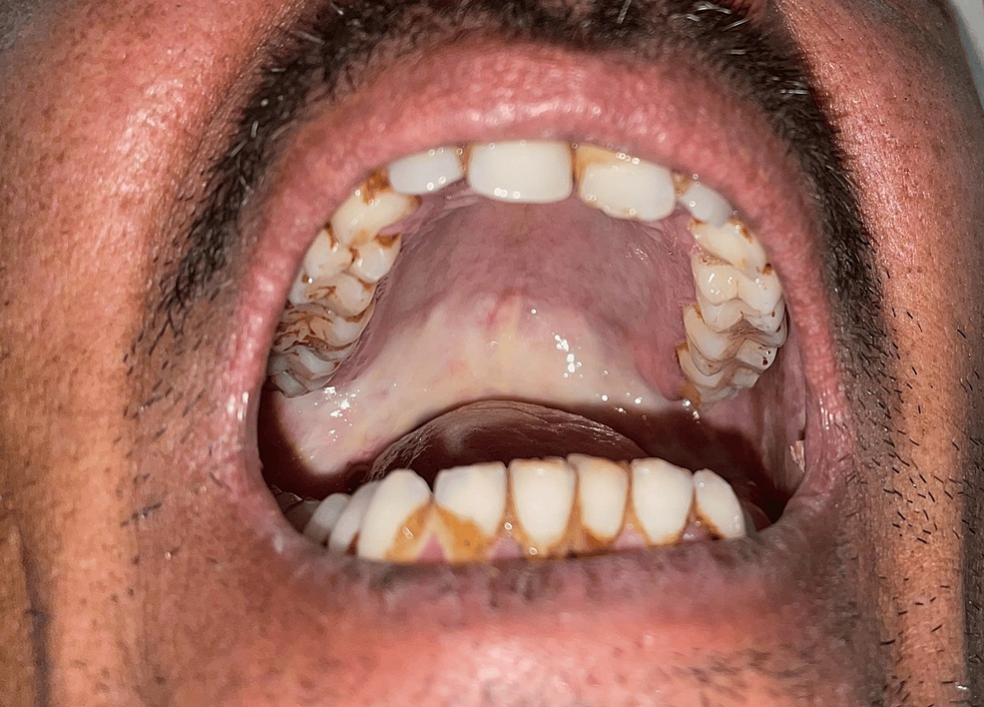
Blanching and stiffness of soft palate and oropharynx. Image of the patient taken by the author.

**Figure 2:**
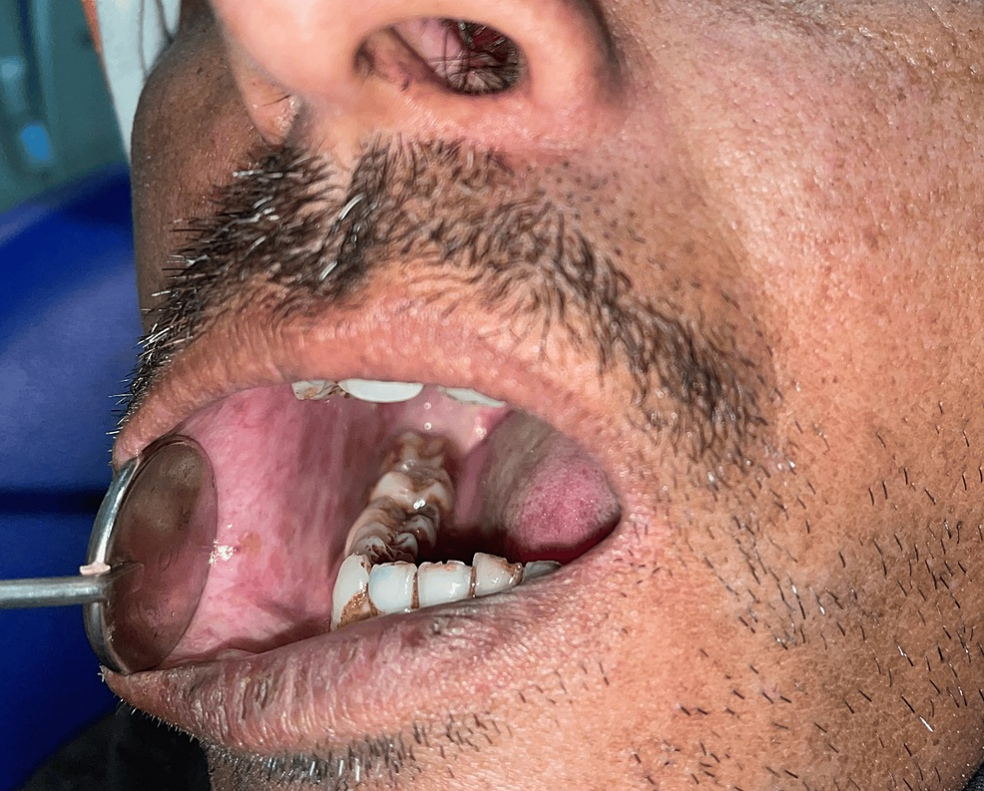
Blanching and stiffness of buccal mucosa. Image of the patient taken by the author.

The precancerous condition known as oral submucous fibrosis (OSMF) affects the submucosa and produces inflammation and increasing fibrosis, which results in significant stiffness and trismus [1]. First identified in the early 1950s, oral submucous fibrosis (OSF) is a potentially cancerous condition that primarily affects people of Asian descent. The clinical manifestation of this chronic, progressive disorder depends on the disease's stage at the time of discovery [2]. The majority of patients initially display a sensitivity to spicy foods as well as lip, tongue, and palate rigidity that, to varying degrees, limits mouth opening and tongue mobility. The hallmark of the illness is submucosal fibrosis, which mostly affects the pharynx, upper third of the oesophagus, and oral cavity. The countries with the highest prevalence of the illness are India, Bangladesh, Sri Lanka, Pakistan, Taiwan, Southern China, Polynesia and Micronesia [3]. Asian immigrants to South and East Africa and the UK have been the subject of several case-series reports. There has been information on a large variance in OSF prevalence among nations. According to recent epidemiological data, the number of patients increased from an estimated 250,000 in 1980 to 2 million in 1993, and OSF cases have increased dramatically in India [4]. The rapid spread of the disease is reportedly due to the increasing popularity of commercially available areca nut preparations (pan masala) in India as well as an increase in young people acquiring this habit as a result of easy availability, successful pricing modifications, and marketing initiatives [5]. With areca nut as the primary aetiological factor, the goal of this study is to evaluate current developments critically to learn more about the pathophysiology and aetiology of OSF. The illness was categorized as an idiopathic sickness when it was first identified in 1952. However, as a possible cause, experts began to point to several environmental agents [6]. Areca nuts, capsaicin in peppers, a lack of micronutrients like iron and zinc, and a lack of vital vitamins have all been mentioned as potential aetiological factors. It has also been suggested that the illness may have an autoimmune aetiology due to the presence of certain autoantibodies and a relationship with particular HLA antigens [7]. This suggests that certain people may have a genetic susceptibility to develop OSF. However, it is evident from the current scientific literature that regular use of areca nuts is the primary aetiological component [8]. The fact that only one publication in literature describes a few examples of OSF developing without any areca nut chewing habit that might be explained by denial of the practice is interesting [9]. A different mechanism linked to undernutrition has been postulated, with atrophy as the underlying cause [10]. This is less likely to be the underlying cause of the disease than to have a confounding effect on it (Figures 1-2).

## Review

Management

**Figure 3:**
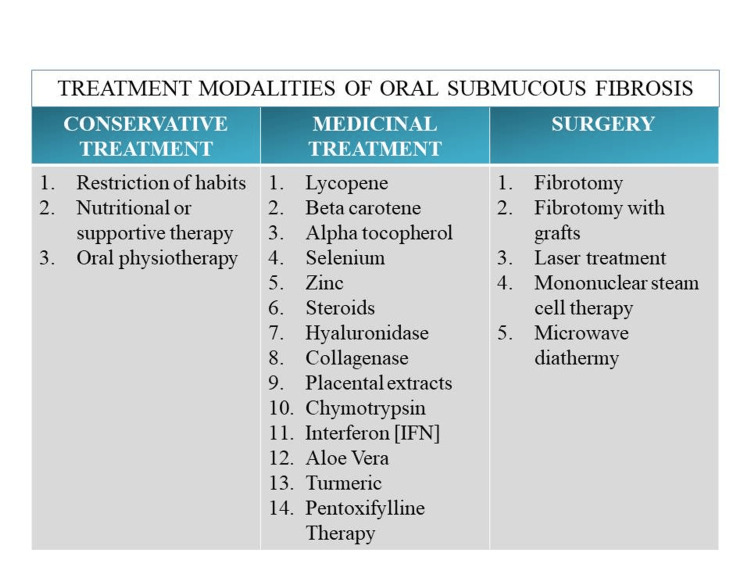
Treatment modalities of oral submucous fibrosis Self-created by the author

The chronic and resistant nature of OSMF is widely established. As OSMF is a premalignant condition and has the potential to change into malignancy, the prime objective of treatment is to arrest the progression of the disease into malignancy [11]. Then symptomatic relief should be given to the patient so that he can improve his dietary status. Several different treatment options are available to treat this illness, including medical approaches, surgical management, and physiotherapy [12]. The first step in proper treatment is educating the patient about the negative consequences of areca nut and related chewing products so that the patient will discontinue the habit. Then the management approaches consist of conservative, medical and surgical management. Along with medical and surgical management, physiotherapy should be prescribed [13]. Treatment modalities of oral submucous fibrosis are in Figure 3.

Conservative treatment 

Restriction of habits, nutritional or supportive therapy, and oral physiotherapy are all examples of conservative treatment.

Restriction of Habits

In India, spices such as gutkha, paan, paan masala, areca nuts, and chillies are consumed. A crucial component of treatment is encouraging the patient to give up the habit at an early stage of OSMF since doing so may help the illness advance more slowly [14].

Nutritional or Supportive Therapy

Micronutrients and minerals including iron, copper, calcium, zinc, magnesium, and selenium, as well as vitamins A, B, C, D, and E, can significantly lower the levels of free radicals. Low fruit and vegetable consumption is associated with a higher risk of developing pre-malignancies and cancers [15]. Since they boost antioxidant levels and provide protection against the increased risk of cancer, fruits and vegetables should be consumed as part of a normal diet. A pigment found in tomatoes called lycopene has been demonstrated to possess several strong antioxidants and anti-carcinogenic properties. It has also been shown to have significant benefits in precancerous lesions like leukoplakia [16]. Green tea polyphenols exhibit significant anti-free radical activity and can shield cells from reactive oxygen species-induced DNA damage. It can also initiate apoptosis and halt the development of malignant cells. Because of this, many of the potential advantages of tea have been associated with the strong antioxidant activity of tea polyphenols. Numerous studies have connected iron deficiency to OSMF's aetiopathogenesis as a cause and a consequence [17]. Therefore, the treatment strategy should include both iron supplementation and routine haemoglobin level tests. A good source of vitamins A, B1, B2, B6, B12, C, pantothenic acid, nicotinic acid, folic acid, iron, copper, and zinc is immune milk, which also has a potent anti-inflammatory impact. IgG antibody treatment could significantly lessen symptoms in OSMF patients by lowering the inflammatory response and changing the cytokine assemblage [18].

Oral Physiotherapy

Exercise programs, splints, and other tools are all forms of physiotherapy that can be used to manage OSMF. To prevent future restrictions on mouth motions, mouth-specific stretching exercises may be helpful; it is thought that this puts pressure on the fibrous bands. Forceful mouth opening has been tried with mouth gags and acrylic surgical screws [19].

Medical treatment

The goal of symptomatic medical care is to facilitate better movement. The following types of medical care can be administered topically, orally, or through submucosal injections.

Lycopene

Lycopene is an example of a carotenoid, which is an organic pigment made from plant sources. Anti-carcinogenic and antioxidant effects are found in carotenoids. Its significant advantages have been shown in precancerous lesions like leukoplakia [20]. Lycopene exhibits aberrant suppression of fibroblasts in OSMF. It controls inflammation response and lymphocyte stress resistance as well. This is the initial course of treatment for OSMF. The largest physical quenching rate constant for singlet oxygen is exhibited by lycopene [21].

Beta-Carotene

Vitamin A is preceded by beta-carotene. It functions as both a radical scavenger and an antioxidant. Free radical damage is reduced by beta-carotene. The immune response is boosted, which slows the growth of cancer cells. T-cells are boosted by beta-carotene, which also boosts the mitogenic response. Administration of beta-carotene increases the amount in the blood, which improves the patient's immune response and slows the growth of cancer cells [22].

Alpha-Lipoic Acid (LA)

Alpha LA is a common antioxidant. It can eliminate free radicals from lipid and water-based media. Hypochlorous acid and hydroxyl radicals are removed by alpha LA, which reduces single oxygen molecules. By promoting the creation of endogenous low molecular weight antioxidants or enzymes and inducing their absorption, alpha LA indirectly maintains the status of cellular antioxidants. Glutathione (GSH), a cellular endogenous antioxidant, is increased by alpha LA. Dietary sources of alpha LA are ingested and accumulate in tissues [23].

Alpha-Tocopherol

Plants contain eight different forms of vitamin E (delta-tocopherol/tocotrienols); however, humans only contain alpha-tocopherol, which has antioxidant properties. It prevents the growth of cells, platelet aggregation, and monocyte adherence as a result of certain interactions with enzymes, structural proteins, lipids, and transcription factors [24].

Selenium

Selenium is necessary for preventing nutritional problems and degenerative diseases. It functions as an anticarcinogenic agent and is found in GSH peroxidase-active sites. It is a nutrient with antioxidant properties that prevent the formation of abnormal cells by inducing methylation of selenium metabolites and selenoproteins. In their paper, Khanna et al. found lower selenium amounts in OSMF patients [25].

Zinc

Multiple cellular processes and improved immunity depend on zinc. Zinc boosts immune system effectiveness by improving the function of neutrophils and natural killer cells, which mediate innate immunity. Zinc enhances the production of cytokines, macrophages, and phagocytosis-induced intercellular killing. Cell division, activation, and RNA transcription all depend on zinc, which also serves to improve the actions and secretions of cytokines. It stabilizes membranes and serves as an antioxidant. In a paper by Ayinampudi et al., zinc levels were found to be 107.13 in OSMF patients against 119.9 in healthy people. This suggested that zinc was being used by the tumour tissues to remedy the zinc shortage [26].

Steroid, Placentrex and Fibrinolytic Drug Injections as Part of Medicinal Treatment

Steroids: Steroids are known to operate as immunosuppressive drugs, inhibiting the inflammation that is present in OSMF lesions and reducing the effects of this fibro-collagenous disease. Additionally, steroids can reduce the number of collagen fibres by inhibiting fibroblast growth [27]. In patients with moderate OSMF, weekly submucosal intra-lesional injections or topical steroids may help prevent further harm. Ulcers and sore oral mucosa may benefit from topically applied steroid ointments; 1.5 cc of hydrocortisone is administered locally and is proven to be effective [27].

Hyaluronidase: Hyaluronidase appears to damage collagen from OSMF patients more quickly than it does normal collagen, according to in vitro research. The hyaluronic acid matrix is broken down by hyaluronidase, which also thins out the intracellular cement and activates specific plasmatic processes. Fibrous tissue is subsequently softened and diminished as a result [28].

Collagenase: One of the factors contributing to the buildup of collagen is the reduced level of functional collagenase seen in OSMF patients. The restoration of eating function is aided by Lin and Lin's discovery; namely, that intra-lesional collagenase injections greatly improve mouth opening while also reducing sensitivities to heat, cold, and spices [29-30].

Placental extracts: Placentrex is an aqueous extract of the human placenta that also contains vitamins, hormones, nucleotides, enzymes, and amino acids. It works by a process known as biogenic stimulation. The tissue therapy technique was first used in 1933. Such tissues, or their extracts, may be implanted or injected into the body after the pathogenic elements have caused conflict in the body. This promotes metabolic or regeneration processes, which is beneficial for healing [31].

Chymotrypsin: The endopeptidase chymotrypsin is an enzyme that can carry out proteolysis [31].

Interferon (IFN)-gamma: Because of its immunoregulatory effects, IFN-gamma is important in the therapy of OSMF. IFN-gamma has been shown to have anti-fibrotic properties, and Haque et al. investigated how it affects the production of collagen by arecoline-stimulated OSMF fibroblasts. In this clinical experiment, intra-lesional injections of IFN gamma significantly improved mouth opening [32].

Aloe vera: The resin, extract, and leaves of aloe vera have antibacterial, anti-inflammatory, and therapeutic qualities. A preliminary investigation was conducted by Sudarshan et al. to compare the effectiveness of aloe vera versus antioxidants in treating OSMF. The results of the study indicate that aloe vera responds more favourably to each parameter evaluated and responds in all clinical-histopathological stages as well, especially in those at mild clinical stages and early histopathological stages of the disease. The burning sensation and the flexibility of the cheeks and mouth both improved with the usage of aloe vera. It was determined that the aloe vera group improves patients' quality of life by reducing the burning feeling and recovering mouth opening [33].

Turmeric: Turmeric contains the naturally occurring yellow pigment curcumin, which has anti-inflammatory, anti-cancer, and antioxidant properties. The potentially cancerous disease known as oral submucous fibrosis (OSMF) is characterized by tight mucosa and restricted mouth opening. Fibrosis causes the oral mucosa and deeper tissues to become rigid, progressively limiting mouth opening and causing tongue protrusion, which makes it difficult to chew, swallow, and phonate [34].

Pentoxifylline therapy: The effects of the methylxanthine derivative pentoxifylline depend on the dose and manifest in the following ways: (1) in addition to decreased platelet aggregation and granulocyte adhesion, the microcirculation is also enhanced; (2) leukocyte deformability is increased, and it also inhibits the adhesion and activation of neutrophils, additionally, it also exhibits fibrinolytic, anti-thrombin, and anti-plasmin action; (3) it can block T-cell and B-cell activation, increase the activity of natural killer cells, and induce neutrophils to degranulate; (4) following an acute injury, it can keep cellular integrity and homeostasis; (5) along with its ability to improve vascularity, this medication can help lessen the symptoms in OSMF patients. Rajendran et al. used pentoxifylline as a supplemental medicine in the treatment of OSMF, which resulted in a seven-month experiment with a 6- to 12-month follow-up period and the patients displayed improvement in their signs and symptoms when compared to the controls [35].

Surgery

When a biopsy reveals dysplastic or neoplastic alterations, it is the preferred approach for patients with limited mouth opening. It includes:

Fibrotomy: Excision of fibrous bands and forced mouth opening are required for the surgical procedure, leaving a raw wound surface. Following surgical relief of the oral trismus brought on by OSMF, relapse is a frequent side effect [36].

Fibrotomy with grafts: To avoid further scarring and trismus recurrence, surgeons started utilizing a variety of interposition graft materials after initially aiming to surgically remove the fibrotic bands. The basic idea is that fibrous bands are cut (incorrectly referred to as "excised") or surgically released, then the mouth is forced open (by widening the incised tissue or region), and surgical deficiencies are covered with a variety of flaps or synthetic biological material [37].

Extra-oral flaps: These include split-thickness skin graft, superficial temporal fascia pedicled flap, nasolabial flap and platysma myocutaneous muscle flap [38].

Intraoral flaps: Flaps are used to treat individuals with severe trismus, and they involve coronoidectomy, temporalis myotomy, split-thickness grafts, and coronoidectomy in addition to the simple removal of fibrotic bands. General anaesthesia is used during the procedure. Due to the mouth's limited aperture, intubation is challenging. The ideal methods for intubating the trachea include employing muscle relaxants with a regional block or deep inhalational anaesthesia. Techniques for guided intubation with fibre optics have also been employed [39].

Laser treatment: Lasers give oral surgeons an innovative method for treating OSMF. With a wavelength of 2780 nm and a high absorption by water, the erbium chromium yttrium scandium gallium garnet (Er Cr: YSGG) laser is utilized to treat oral soft tissue without causing thermal harm [40]. The advantages of laser surgery as a whole include a largely blood-free operating area, which enhances visibility, a decreased need for local anaesthetic, a smaller risk of bacterial infection, less mechanical tissue stress, fewer sutures, quicker healing and a decreased incidence of postoperative swelling, scarring, and tissue shrinkage. During follow-up, while discussing an effort to treat a minor case of bilateral OSMF with Er Cr, the YSGG laser achieved superior outcomes [41].

Mononuclear stem cell therapy: The effectiveness of stem cell therapy in the treatment of OSMF was examined in a 2013 study by Seshadri et al., who assessed the improvement in function and the sustainability of the result with a five-year follow-up [42]. Three of the seven patients received stem cell treatments using the ficol method, while four received stem cell treatments using point-of-care delivery systems [43]. Histopathological characteristics evaluated and corroborated the clinical presentation's post-treatment improvement. Cases were followed up at any time between six months and five years [44]. The mucosa became more flexible, the burning feeling brought on by consuming spicy food subsided, and mouth opening increased. During the observational period, it was found that the subsequent improvements had endured [45].

Microwave diathermy: Heat treatment works by causing band fibrinolysis. The region that can be treated is constrained by microwave diathermy, which only heats juxta-epithelial connective tissue. It is therefore simple to apply and causes little irritation [46].

## Conclusions

There are numerous management strategies for OSMF, according to this survey of the literature on the subject. Corticosteroids, hyaluronidase, placentrex, IFN, and microwave diathermy, among other therapy modalities, have all been linked to the ability to treat the illness. A surgical procedure that involves cutting away fibrotic tissues & filling the defect with grafts is also being studied. However, as of right now, there isn't a single technique that can be employed as the sole form of OSMF treatment. More in-depth clinical trials with more cases and more parameters are required to make a solid determination about a certain modality for treating OSMF. Recent research has shown that the pharmacological combination generates beneficial effects in the management of this condition.
